# The COVID-19 outbreak in Iran

**DOI:** 10.7189/jogh.10.010365

**Published:** 2020-06

**Authors:** Rasoul Salimi, Reza Gomar, Bahram Heshmati

**Affiliations:** 1 Emergency Department, Besat Hospital, Hamadan University of Medical Sciences, Hamadan, Iran; 2 Emergency Medical Services, Emergency Department, Kermanshah University of Medical Sciences, Kermanshah, Iran; 3 Department of Medical Journalism, School of Paramedical Sciences, Shiraz University of Medical Sciences, Shiraz, Iran

According to the World Health Organization, as of April 21, 2020, 2 397 217 cases of coronavirus disease 2019 (COVID-19), including 162 956 deaths, have been reported worldwide [1]. The outbreak began in Iran after the detection of the first death associated with COVID-19, on Feb 19, 2020 in Qom, a holy city in central Iran. After a short period, COVID-19 has widely spread in all other provinces in Iran. As of April 21, 2020, of 330 137 tested patients, 80 868 people have been infected with COVID-19. Of them, 55 987 people have recovered, 3513 people are critically ill and 5031 people have died [2].

The formal announcement of the outbreak in Iran has generated public panic and anxiety. The sudden explosion in the number of suspected cases of COVID-19 in the first week in several provinces has overwhelmed some designated hospitals very quickly. Medical personnel faced shortages of protective equipment, essential medications and care facilities. People rushed to the stores to purchase masks, gloves and disinfectants. This created a black market, which some hospitals had to rely on to provide the protective equipment. Furthermore, fake news and misinformation have increased public anxiety. To respond to the outbreak, the Headquarter for coronavirus combat and prevention has been established. The main measures, such as stopping mass gatherings, closure of educational institutes, national coordination with volunteers, civilian and military forces, national screening program, and social distancing led to shortage management to some extent. It had a potential to alleviate some of the public fear. Although the measures that were implemented to control the outbreak, along with some half-measures, may have made it harder to control the disease. At the same time, they could affect the economy through long term and avoidable burden of COVID-19. Also, there was a question whether early stopping of the mass gatherings such as Parliament Election and restrict travel in Qom could have acted to slow the spread of the disease through Qom and into other cities.

Measures like the closure of schools, educational institutes, religious and sacred places (despite the opposition of some religious figures), and stopping mass gatherings - such as religious and sporting events - were the major pillar of the response to the outbreak. They caused reduction in the number of COVID-19 super-spreading events in Iran. Authorities widely reminded the public of their role to control the outbreak. They strongly encouraged people to stay home and avoid social contact with family and friends. Before the Iranian New Year Festival, people were urged to strongly avoid familial gatherings and trips to celebrate the New Year. Most of the people followed these recommendations, but some ignored them. After the New Year, on the 20th of March, 2020, some trips did occur and some people left the cities to visit their hometowns. Although travelers were screened with thermal scanners at the points of entry and exit to those cities, this was not a guarantee that the possible importation of the disease to other cities and rural areas would be prevented because fever is not always a symptom. As a consequence, stricter measures, especially travel bans, were introduced on March 26, 2020. The measures had the desired effect and lead to flattening the epidemic curve. However, is seems that New Year holidays missed due to avoidable delays and the question remained - if stricter measures were launched before the new year, could they have been more effective in the control of the outbreak?

As one of the main measures, the national screening program was implemented on March 17, 2020. It was set up to allow early detection, diagnosis, isolation, and treatment of the new cases, and to follow their contacts. The screening was performed by primary care providers or people themselves via website (https://salamat.gov.ir/) or a phone number (4030). Screening was based on the questions about the symptoms of COVID-19. Suspected people were referred to special 16-hour medical centers and screened by the COVID-19 response teams based on body temperature, Spo2, respiratory rate, lung scanning (if available) and COVID-19 diagnostic tests. Subsequently, screened patients were isolated at home, at isolation centers or hospitalized in designated hospitals (**Figure 1**). This measure significantly reduced the massive influx of infectious cases to emergency departments and hospitals. But without widespread testing, the screening could not be so helpful in cutting down the transmission chain.

**Figure 1:**
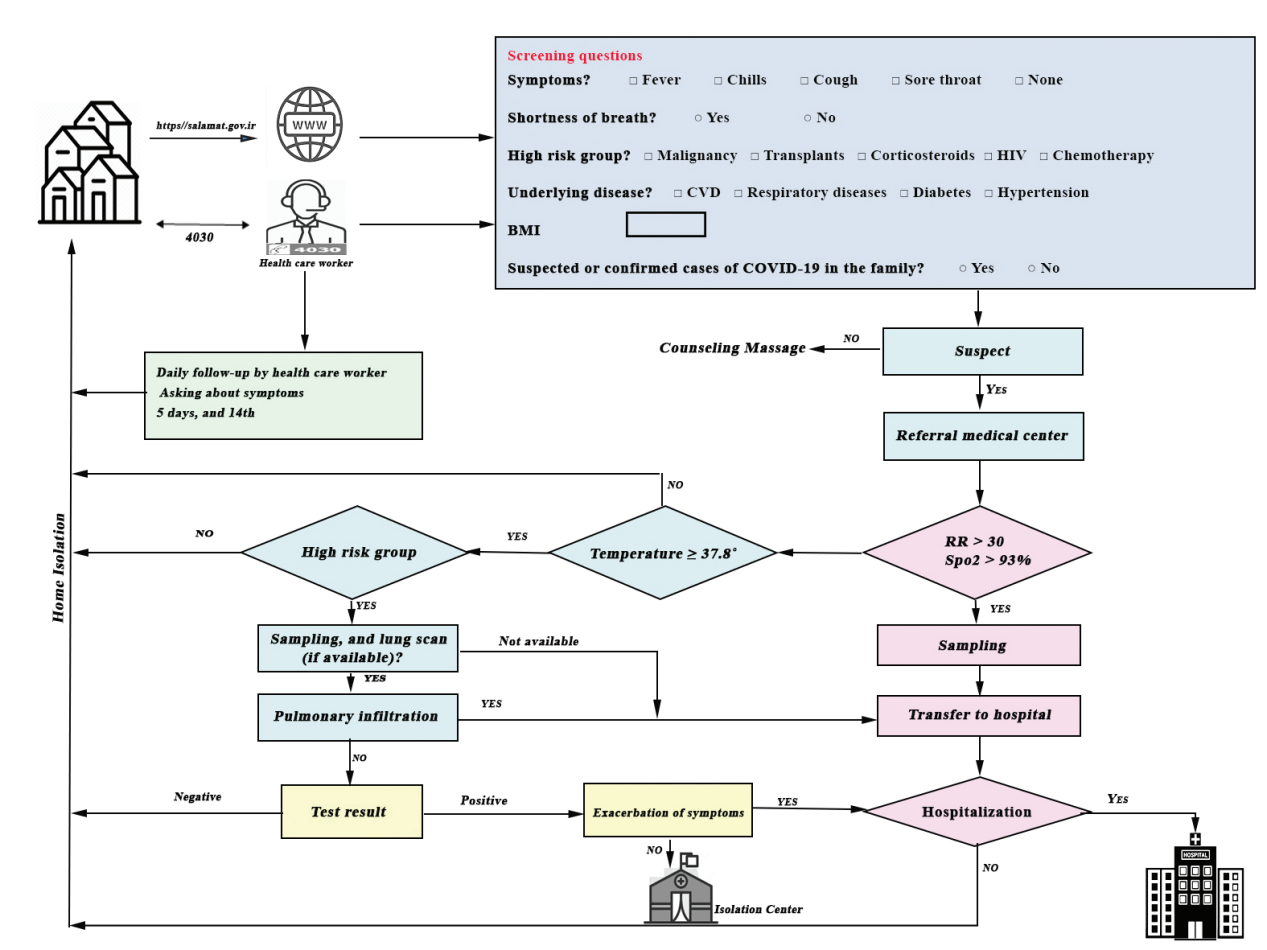
The Iran national screening program algorithm of response to the COVID-19 outbreak (started on March 17, 2020). Screening is done by primary care providers or people themselves via a website or a phone number based on screening questions. Suspected cases undergo further evaluations by the COVID-19 response team in the special 16-hour medical center. Based on body temperature, peripheral capillary oxygen saturation (SpO2), respiratory rate, lung scanning (if available), and COVID-19 diagnostic test, people isolate at home, isolation centers or become hospitalized in designated hospitals.

The response to COVID-19 outbreak in Iran faces some challenges. Two of the main challenges that suffered painfully during the outbreak were sanctions against Iran and the circulating fake news and misinformation on social media networks. The US unilateral economic sanctions against Iran and the further recent sanctionary measures [3] have restricted the import of essential goods. Successful actions in stopping the outbreak required sufficient essential medicines and facilities. So, it was critical that sanctions and barriers to providing essential supplies to Iran would be stopped, or to be postponed at least until the end of the COVID-19 epidemic. On the other hand, fake news and misinformation imposed hardship on the health system. A fake news, “drinking alcohol has a protective role for COVID-19” disseminated through multiple social media channels, caused more than 3000 cases of poisoning and more than 700 deaths associated with drinking fake alcohol to date [4]. Effective communication will help eliminate fake news and promotion of appropriate behavior. So, authorities should pay more attention to social media networks.

**Figure:**
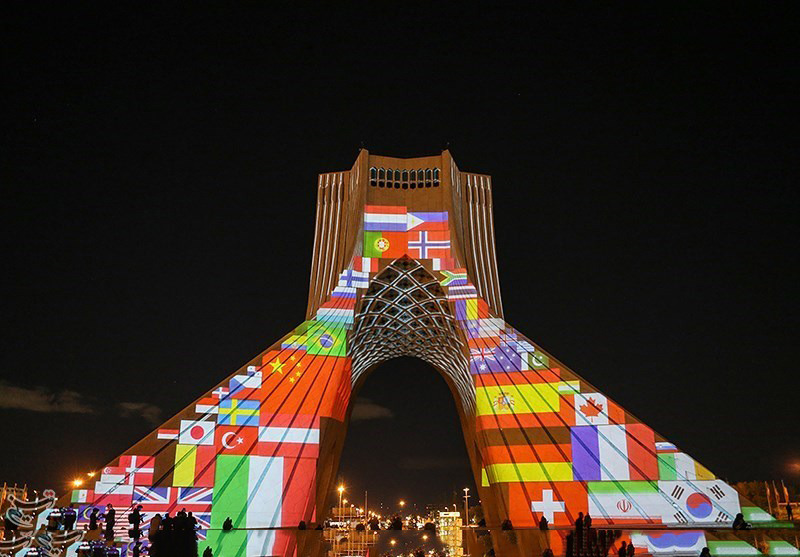
Photo: The video-mapping performance staged at Tehran’s Azadi Tower to show the unity of Iranians with world countries in the fight against COVID-19. (Source: Tasnim News Agency, licensed under CC BY-SA 4.0; available at https://www.tasnimnews.com/fa/news/1399/01/12/2234302/).

## CONCLUSION

All interventions during the outbreak helped to flatten the curve of COVID-19 cases in Iran. With some half-measures, control of the outbreak will prolongate and new waves will be expected. Some aspects of the response to the outbreak need more attention. First, the number of diagnostic tests is very low. Therefore, the scope of the outbreak in Iran cannot be traced. Widespread testing must be performed to interrupt new transmission chains and keep clusters under control. Second, early reduction of the restrictions by the government to avoid severe economic impact, along with the fact that the prolonged duration of staying home had put many people at risk of depression and anxiety, can both lead to increased risk of premature end to restrictions. This can cause further waves of infection with varying intensity and duration. Third, the government has promised people financial support. Some promises have been postponed. Delay to receiving timely support might lead to more stress and anxiety and can prevent effective action. Finally, medical facilities shortages, prolonged disease outbreak, and the further waves of infection can increase the mortality risk among health care workers, more reporting of physical and mental exhaustion, irritability, poor work performance, reluctance to work, and burnout.
